# Preschool teacher attachment and attention skills

**DOI:** 10.1186/2193-1801-2-673

**Published:** 2013-12-16

**Authors:** Elena Commodari

**Affiliations:** Department of Educational Processes, Unversity of Catania, Catania, 95127 Italy

**Keywords:** Attention skills, Attachment, Children, Preschool

## Abstract

Attention underlies and energizes all cognitive and behavioral activities. Many studies showed that the quality of child attachment (both to parental and non parental figures) influences cognitive functions and attention. This study aimed to investigate the relationships among attachment to preschool teachers and attention in a sample of preschoolers. In particular, the study analyzed whether child attachment security to preschool teachers influences the different aspects of their attention skills. In addition, gender- and age-related differences in attention and teacher attachment were explored. Research was conducted using two standardized instruments: the Attention and Concentration Battery, and the Attachment Q Sort. Participants were 279 children (147 male, 132 female) who attended two preschools in a town in Southern Italy. Descriptive analyses, *t*-tests analyses, and correlation and regression analyses were carried out. Findings highlighted several interesting points concerning the relationships that occur among attachment to preschool teachers and attention. Children with secure attachments presented higher reaction time and better auditory, visual, and visual spatial selectivity and maintenance.

Cognition is the information-handling aspect of behaviour. It comprises four classes of processes, “receptive functions”, “learning and memory”, “thinking” and “expressive functions” that are the common background of all complex performances (Lezak 1995). To these four groups of processes may be added “attention”. Attention differs from the other four classes of processes because it underlies and energizes the activity of all cognitive functions.

Attention comprises several different aspects, such as “reaction time”, “selectivity”, “maintenance”, “voluntary shifting”, and “divided attention”. These aspects are involved in learning, memory, and programming of both motor and behavioural responses. Attention permits to detect the appearance of the environmental stimuli, and influences the capacity to highlight a stimulus while suppressing awareness of competing distractions (Johnston and Dark 1986; Russell 1975). Moreover, it determines how much information can be grasped and maintained at the same time (Douglas 1979; Howieson and Lezak 1994), and the capacity to respond to more to than one task at a time or to multiple elements or operation within a task as in a complex mental task (Sohlberg and Mateer 1989; Stuus et al. 1989). Finally, attention is involved in the capacity to shift in focus and tasks (Sack and Rice 1974; Sohlberg and Mateer 1989).

Several studies (e.g. Garon et al. 2008; Rothbart and Posner 2001; Raver et al. 2005) have evidenced that attention provides the foundation for developmental gains in all other cognitive functions during the preschool years. Moreover, disturbances in attention are implicated in most of the psychological disorders. Attentional difficulties have been linked to many cognitive and behavioral deficits, such as impairment in executive functions, impulsive behavioral style (Raaijmakers et al. 2008), academic failure, difficulties in basic scholastic skills, and learning difficulties (Duncan et al. 2007; Robinson and Winner 1998; and Scanlon, Vellutino et al. Vellutino et al. 2004; Howse et al. 2003; Yen et al. 2004). Moreover, sustained attention and disinhibition are empirically related during infancy and toddlerhood (Kochanska et al. 2000), as well as during the school years and adulthood (Barkley 1997; Berlin et al. 2003).

## Attention and attachment

Several researches have analyzed social and environmental factors that influence attention (Fearon and Belsky 2004). Considering that stress affects a wide range of cognitive processes, particularly attention and memory (Lupien and McEwen, 1997; Mendl 1999), some of these studies have investigated the relationship between stress and children’s attentional performance. Other studies analysed the relationships between the quality of the child bond with their caregivers and attention functioning and explored the influence of child/caregiver attachment on attentional performances (Davis, Bruce, and Gunnar, 2002; Lupien and McEwen, 1997; Skosnik et al. 2000; Wolkowitz et al. 1990; Wolkowitz et al. 1993).

Attachment is the emotional bond between children and their caregivers (parents or other figures taking care of them). The child uses the attachment figure as a secure base from which to explore the environment and a safe haven in times of distress. The formation of attachment to caregivers is a normative event; all children form attachments to their caregivers even if they do not receive adequate care, and thus attachments vary in quality.

Different attachment patterns (secure, anxious-ambivalent, anxious-avoidant, disorganized-disoriented) have been identified (Ainsworth and Bell 1970; Main and Solomon 1990). “Secure” children use the mother as a secure base for exploration and seek contact with her after a separation; “anxious-ambivalent” children are unable to use the mother as a secure base and are often angry and push her away upon reunion after a separation; “anxious-avoidant” children fail to use the mother as a secure base for exploration and avoid the mother upon reunion or approach her only indirectly; “disorganized-disoriented” children have no predictable or effective pattern of eliciting care-giving behaviors when stressed.

Attachment security influences cognitive development (Cassidy 1988; Marvin and Britner 1999; Vandell et al. 2010). Many studies reported security related differences in language acquisition, symbolic play and deductive reasoning. Moreover, children with insecure disorganized attachment representations were likely to be strongly inhibited from engaging in cognitive transactions with their environment (Matas et al. 1978; Jacobsen et al. 1994). In addition, some researchers have discovered that secure child attachment promotes positive outcomes including prosocial beliefs (Catalano et al. 1996), self esteem and higher levels of social competence (Greenberg et al. 1983; Rice et al. 1997).

With regards to attention skills, several studies evidenced that secure preschoolers tend to have higher attention skills and develop better scholastic skills, such as reading/pre-reading skills and attitudes toward reading, compared with insecurely attached preschoolers (Bergin and Bergin 2009; Bus et al. 1997; Bus and van Ijzendoorn 1988; Frankel and Bates, 1990; Moss and St-Laurent 2001). Main’s (1983) early observational study showed that 3- year-olds with secure attachment histories manifested longer attention spans during play. Secure attachment may also promote the development of attentional control because of the role sensitive parenting plays in fostering attachment security (De Wolff and van IJzendoorn, 1997). With regards to this, (Ruff and Rothbart, 1996) have suggested that attention is influenced by parenting processes. This proposition is supported by a recent work, showing that maternal sensitivity, measured repeatedly across the first 4–5 years of life, predicted attention at age 54 months, even after controlling for family background variables (NICHD Early Child Care Research Network, 2003).

Anxious attachment is linked to ADHD. Insecure children are more likely to be diagnosed with ADHD, or have ADHD symptoms regardless of being diagnosed (Clarke, Barry, R.J., McCarthy, Selikowitz, and Brown, Clarke et al. 2002; Egeland et al., 1993). A study involving 5 to 7 year olds children with disorganized attachment found they had social and attention problems according to their teachers (Goldwyn et al. 2000). Presumably, anxiety impairs ability to control attention, executive functions, memory and problem-solving, and increases task-irrelevant thoughts (Fincham, Hokada, and Sanders, Fincham et al. 1989; Ialongo, Lopez, Horn, Pascoe, and Greenberg, Ialongo et al. 1994). Moreover, (Fearon and Belsky 2004) found that children with secure attachment were less susceptible to the effects of cumulative risk and gender on attentional performance than their insecure counterparts. The link between attachment security and ADHD symptoms may be due to its effect on emotion regulation and anxiety.

### Attachment to preschool teachers

Infants and young children usually have more than one selective attachment (Rutter and O’Connor 1999). Most children form a network of attachment relationships with the family and other people who take care of them (including those between children and teachers). These relationships have an important effect on cognitive and social acquisitions (DeMulder, Dehnam, Schmidt, and Mitchell 2000; Howes 1997; Howes and Smith 1995; Mitchell-Copeland et al. 1997; Rutter and O’Connor 1999; Seibert and Kerns 2009). Several authors have investigated child attachment to teachers (Birch and Ladd 1997; Beishuizzen et al. 2001; Hamre and Pianta, 2001; Howes and Ritchie 1999; Pianta et al. 1997; Pianta and Nimetz 1991).

(Howes and Ritchie 1999) described four types of attachment to teachers that parallel the typology of parent/child attachments, and there is an increasing interest in child/teacher relationships as a predictor of their cognitive and educational competence. Young children who develop secure relationships with their teachers, often feel more confident in the caring environment, have enhanced cognitive activity and will be more successful at learning in that environment (Bergin and Bergin 2009; Birch and Ladd 1997; Pianta 1999). (Howes et al. 1998) provided evidence of continuity in the relationship between child-teacher attachment and learning in a longitudinal study. More recently, (Mashburn et al. 2008) examined the development of academic, language, and social skills among four year-olds in pre-kindergarten programs. They found that improved teacher/child interactions facilitate school readiness.

### Aim of the research

Attentional processes underlie and energize all cognitive and behavioral activities. Many studies showed that the quality of child attachment (both to parental and non parental figures) influences cognitive functions and attention. On the basis of these considerations, the overall goal of this study was to investigate the relationships among attachment to preschool teachers and attention functioning in a sample of preschoolers. In particular, this study seeks to provide a better understanding of the role of attachment security in the different aspect of the attentional performances. In addition, gender and age-related differences in attention and teacher attachment in preschoolers were explored.

## Methods

### Participants

Research was conducted using a sample of 279 children (147 male, 132 female) who attended two preschools in a town in Southern Italy. Unlike the USA in which kindergarten and preschool are separate (i.e., American children attend preschool from 3 to 5 years and kindergarten at 5–6 years because the latter curriculum is a part of the public school curriculum), Italian children attend the same childcare setting (called “preschool” in this study) from 3 to 5 years, and begin primary school at 6 year of age. Preschool teaches children the prerequisite skills of reading and writing but not the ability to read and write.

All participants were between 4–5 years old, come from two-parent, intact families, and attended the preschool where the research was conducted for at least one year. 140 children were four years old, and 139 children were five years old (mean age = 4.50, *SD* = .52). Participants spent at least 25 hours per week at the preschool from Monday to Friday. The teachers (mean age = 39.2, *SD* = 4.3) who cared for the children had a specific education certificate (high school certificate) and the post-certificate qualification required by Italian law. The ratio of teachers to children was one to 15. All of the teachers were female.

### Measures

This study was conducted using two standardized instruments: the Attention and Concentration Battery (Di Nuovo, 2000) assessing attention skills, and the Attachment Q Sort (AQS, Italian version, form evaluating child/teacher attachment, Cassibba and D’Odorico 2000; Waters 1987) evaluating child attachment. The Attention and Concentration Battery (Di Nuovo 2000) is a computerized battery involving seven different tests. It assesses visual reaction time to unselected stimuli, visual reaction time related with a choice, visual, visual-spatial and auditory selectivity, sustained attention, digit span, divided attention, resistance to distraction, and attentional shifting.

The first test, “Simple Reaction Time,” measures the visual reaction time to unselected stimuli. The stimulus was a star, presented for 50 msc. at the centre of the screen, preceded by a cue stimulus to direct the fixation. The interval between the appearances of two consecutive stimuli was randomized (average 1.5 sec.). After a training section the child is presented a stimulus. He is instructed to push, used the favoured hand, a specific computer key following a visual stimulus. The score is established by evaluating the number of correct answers (correct responses score) and the median reaction time for 30 trials (response times score). The range score was 0–30.

The second test, “Speed and Accuracy,” measures the reaction time related with a choice. The task measures speed and accuracy of response to complex stimuli and requires pushing the key correspondent to one of a set of stimuli. The stimuli are numbers (from 1 to 7) presented at the centre of the screen in randomized sets (from 3 to 5 elements). In each trial of the task, one number of the set appears ringed round. The child is instructed to push a key correspondent to the ringed round number. The scores are established by evaluating the number of correct answers (correct responses score, range scores: 0–30) and median time of answer (response time score).

The third test includes auditory and visual tasks and measures selectivity and maintenance on auditory, visual and visual spatial dimensions. The “Auditory Recognition” test measures auditory selectivity and requires recognizing auditory target among vocal distractors. This task consists of pushing a particular key following a vocal stimulus. The stimuli are letters (vocals and consonants). The target is letter “o”. The scores are established by evaluating the number of correct answers (correct responses score, range scores: 0–15) and median time of answer (response time score).

The “Visual Recognition” test includes two different tasks. The first, “Visual Recognition” task, requires recognizing a visual target among group of distractors appearing in sequence. The stimuli are images of common objects (for example umbrella, star, fruit). The child is required to push a key when a star appears on the screen. The scores are the number of correct answers (correct responses score, ranges score: 0–15) and median time of answer (response time score). The second, “Visual-Spatial Selectivity” task, is a computerized version of symbols barrage test. The child has to delete a particular symbol of a set of stimuli. In the screen a set of stimuli appears and each stimulus is sequentially ringed round. The subject has to push a particular key when the target is circled. The range scores is: 0–15.

The fourth test, “Digit Span,” is an adjustment of the classic Digit Span. It comprises, as in well-known Wechsler Scale subtest, two different tasks: digit forward, and digit backward. The task requires repeating each digit sequence exactly as it is given in forward test, in reversed order in backward test. The tasks measure a simple immediate attention span. The range scores is 0-9- The fifth test evaluates divided attention by a double contemporaneous task (visual recognition and auditory recognition, range scores: 0–15 for each task).

The sixth test, “Colour Word Interference Task,” measures selectivity and the capacity to inhibit interference of non pertinent signals. It is an adjustment of classic Stroop test (1935) that requires the naming of the colour of ink used to print a word describing a different color (for example “red” written using blue ink), overcoming the interference originating from the habit of reading the word. The computerized adaptation of this test consists of two sequential tasks, the first, base line task, used as baseline and the second, interference task, as interference test. In this study this test was not used, considering that participants were not able to read.

The seventh test, “Attention Shifting” test, measures the attentional shifting through two search tasks, one of verbal symbols and one of visual-spatial symbols. For each task the scores are established by evaluating correct answers (correct responses score; range score: 0–90) and median time of answer (response time score).

The Attention and Concentration battery was administered in individual sessions, in a computer laboratory by a trained observer. The tests were administrated in a random order. Each task was preceded by a training session, regardless of whether children had previous experience using computers. Attention and Concentration battery supplies a computerized scores system. The battery has emerged as a psychometrically sound procedure to measure attention. Reliability test- retest of all battery at an interval of 15–20 days (*r* values comprised between .82 and .92 for the different tests) and concurrent validity (*r* values comprised between .80 and .90 for the different tests) are satisfactory (Di Nuovo 2000).

The AQS analyzes attachment to the mother/professional caregiver. In this study, the Italian version (form evaluating child-teacher attachment) of the AQS was used. The AQS items describe the secure-base behaviour of one- to five-year-olds at home or at indoor/outdoor public places. Version 3.0 of the AQS consists of 90 statements that describe a young child's behaviour during caregiver interactions. Its items were designed to provide a comprehensive description of “secure-base” behaviour with caregivers. Similar to other Q-sorts, the AQS is performed by sorting the 90 items into categories using a fixed distribution. An attachment security score is derived by comparing the resulting descriptions with the behavioural profile of a prototypical secure child as provided by several attachment theory experts. The AQS can be sorted by trained observers or by the attachment figure who is being assessed (e.g., the mother, father, or teacher).

(Waters and Deane 1985) extensively discussed the items content and sorting procedure of the AQS and concluded that both are appropriate to measure attachment; thus, this assessment has content validity. Moreover, many studies have shown that the AQS is a valid measure of caregiver/child attachment. Test-retest reliability, inter-observer agreement, and predictive, convergent, and discriminant validity are satisfactory (Cassibba and D’Odorico 2000; DeMulder et al. 2000; Denham and Burton, 2003; Goossens and van Ijzendoorn, 1990; Moss, Bureau, Cyr, and Dubois-Comtois, Moss et al. 2006; Teti and Mc Gourty, 1996; Van Ijzendoorn et al., 2004).

Several adjustments to the number of items and phrasing of the AQS have been developed. (Van Ijzendoorn et al. 2004) found the AQS has shown to be robust against these minor adaptations.

The current study uses the Italian form to evaluate attachment to professional caregivers (Cassiba and D’odorico, Cassibba and D’Odorico 2000). It was derived from the Italian version of the original AQS and assesses the infant/caregiver attachment in the context of childcare centers. This version is similar to the original one but replaces the terms “mother” and “home” with “caregiver” and “childcare centers,” respectively.

With regard to the psychometric characteristics of this AQS form, the test-retest reliability Pearson *r* = .83) and inter-observer agreement Pearson *r* = .70) were satisfactory (Cassibba, Van Ijzendoorn and D’Odorico 2000; Cassibba et al. 2000). The level of inter-observer agreement was lower but not significantly different from the one obtained for maternal attachment.

In the present study, a trained observer, with previous experience with this AQS form, sorted the AQS. The preschools in which the data were collected presented typical structural, and organizational characteristics of public preschools in Italy. The observer visited children and their caregivers at preschool twice in one week. During observation periods, the teachers were encouraged to perform routine classroom activities while the observer monitored the children. A second observation was scheduled two - three days after the end of the first visit. The observer sorted the AQS at the conclusion of the second observation. Each visit lasted at least three hours. A previous meta-analysis showed that AQS data that are more valid have been collected in studies with more than three hours of observation (Van Ijzendoorn et al. 2004).

Criterion sort scoring, in which experts use a q-set to define a construct and compare that description of individual participants to the q-set defined hypothetically most secure children (i.e., the criterion), was used. The similarity between this criterion sort (an array of n-item means) and the q-sort description of a particular participant (either an array of n scores from one observer or the average of several observers) is used as the participant’s attachment score. This similarity is usually measured by correlating the n-item array of criterion sort scores with the n-item array of scores that describe the participant. These correlation coefficients are used as the participants' scores on the construct.

Because the AQS criterion sort scores are correlation coefficients (i.e., *r* scores), these scores were converted to *z* scores using Fisher's *r* to *z* transformation to increase their linearity. The sampling distribution of Pearson’s *r* is not normal. (Waters 1987) recommended this transformation and even suggested it for the specific AQS version used in this study (Cassibba and D’Odorico 2000; Reynolds and Miller 2003). The Italian version of the AQS includes a computerized scoring system.

### Procedures

Trained observers monitored the children for a prolonged time in their classroom. Tests were administered in a familiar, well-known setting at the preschool away from distracting noises. The trained observers were women with university degrees in “infancy education” and specific training on the observation techniques of child behavior. Moreover, these observers had previous experience with the observational measure used in this study. Before beginning data collection, observers participated in a training session to familiarize themselves with the procedures. Two observers systematically and independently observed each child. The agreements between the AQS observers were satisfactory (r = .79; *p* < .01). The sequence of observations and test administration were counterbalanced by all the participants. Institutional review approval by school office was obtained, and parental consent for each child was obtained.

## Results

First, descriptive analyses and *t*-tests examined differences in the means with respect to sex and age; second, *t-*tests using the AQS security scores as an independent variable and either Attention and Concentration Battery scores as dependent variables were also carried out. Correlation and regression analyses examined the relationships among the major variables.

### Descriptive analyses

Table 1 shows the descriptive analyses of the Attention and Concentration and AQS scores including the means and standard deviations of all measures by sex and age. Boys outperformed girls on the following Attention and Concentration tests: “reaction times” (response time scores, *t*: -2.8, *p* = .006, df: 137) and “visual spatial recognition” (correct responses scores; *t*: 2.12, *p* = .03, df: 134.1). Present data evidenced that sex differences in attention skills appeared precociously in the life span. This finding is consistent with previous studies, showing that males and females differed in some aspects of attention, such as selective attention (Merrit et al., Merritt et al. 2007), visual-spatial attention (Collins and Kimura 1997, Commodari, 2012) and sustained attention (Giambra and Quilter 1989). Sex differences were observed also in the AQS scores (*t*: 3.93, sig: *p <* .001, df: 116.1). These results were similar to that obtained by Howes and Smith (1995a) in a study on attachment to non parental figures. Authors found that girls were more likely to form secure relationships with their care providers than boys were.

**Table 1:** **Descriptive analyses of the measures including the means and SD by gender and age**

	All Sample		Male		Female			Aged 4		Aged 5		
Measures	***M***	***SD***	***M***	***SD***	***M***	***SD***	***t***	***M***	***SD***	***M***	***SD***	***t***
**AQS scores**	.46	.14	.42	.15	.51	.11	3.93*	.46	15.91	.46	.12	.06
Attention & concentration												
**Reaction time**												
Correct	28.	4.2	28.45	5.05	27.95	3.08	.69	28.05	3.08	28.36	5.11	-.42
Time	.42	.25	.36	.25	.48	.24	-2.8*	.39	.28	.45	.21	-1.37
**Speed and accuracy**												
Correct	7.53	9.27	8.42	10.32	6.60	8.03	1.15	.40	2.34	14.77	7.97	-14.44*
Time	.71	.75	.72	.74	.70	.76	.16	.05	2.77	1.38	.41	-22.43*
**Auditory recognion**												
Correct	7.40	1.72	7.63	1.39	7.15	1.99	1.67	6.77	1.81	8.13	1.25	-5.49*
Time	.92	.31	.96	.28	.88	.33	1.59	1.01	.36	.83	.21	- 3.51*
**Visual recognition**												
Correct	7.73	1.8	7.62	1.97	7.85	1.74	-.73	7.14	2.3	8.33	.92	-3.97*
Time	.74	.22	.74	.25	.75	.19	.31	.82	.23	.67	.17	4.38*
**Visual spatial recognition**												
Correct	8.57	2.7	9.04	2.54	8.07	2.85	2.12*	7.34	2.72	9.81	2.08	-5.98*
Time	.63	.20	.62	.20	.63	.20	-.40	.69	.19	.56	.19	3.94*
**Digit span**												
Forward	1.23	3.1	.10	.1.17	1.57	4.32	1.26	.00	.00	2.48	4.11	-5.04*
Backward	.34	.70	..45	.85	.22	.48	1.93	.68	.88	.00	.00	-6.45*
**Divided attention**												
Correct	5.52	2.62	5.66	2.60	5.37	2.61	.66	4.11	2.65	6.94	1.65	.31
Time	.92	.40	.91	.35	.93	.45	-.30	.98	.50	.87	.26	-.75
**Multiple search**												
Letter – Correct	6.15	2.84	6.39	2.50	5.90	3.09	1.03	5.04	3.03	7.28	2.12	.6.15*
Letter – Time	115.4	68.27	130.73	70.40	99.43	62.56	2.76*	131.03	78.68	99.53	51.69	2.78*
Simbol – Correct	5.65	3.06	5.73	3.17	5.56	.29	.33	4.69	3.34	6.62	2.41	-3.91*
Simbol – Time	125.0	70.06	127.65	70.08	122.34	70.45	.44	138.53	81.94	11.36	52.63	2.32**

Note: correct: correct answers score; time: response time score. *p < .05. **p < .001.

Significant age related differences were observed for the most of the “Attention and Concentration” battery scores (sig.: *p* < .001). These findings were expected, considering the developmental characteristics of attention skills. There were no significant differences in the AQS criterion sort scores between children aged 4 and children aged 5.

### Mean comparisons with AQS security as an independent variable and either attention and concentration scores as the dependent variables

The sample was divided into two groups based the AQS criterion sort scores. Following (Howes et al. 1990) and (Ahnert et al. 2006), the continuous AQS measures were converted into categories. Children with AQS values > .33 were deemed as “more securely attached”; those with AQS values < .33 were deemed as “less insecurely attached” Among all participants, 226 children were classified as having more secure attachments, and 53 children were classified as having less secure attachments. Children who were considered more securely attached showed better performances on “reaction times” (time score: *t:* 2.94*, p* = .004), “auditory recognition” (correct answers score: *t* = 2.08, sig.: *p* < . 0.39; time score: *t* = -3.67, *p* < . 001), “visual recognition” (correct answers score: *t* = 2.76, sig. *p* = .006), “visual spatial recognition” (correct answers score: *t* : 2. 42, sig : *p* = .01), “divided attention” (correct answers score, *t* = 3.77, sig: *p* < .001),and “multiple search” (letter: time score, *t:* 4.02, *sig: p* < .001; symbol: time score, *t*: -3.07, sig: *p* < .001) tests compared with less securely attached children (Table 2).

**Table 2:** **Mean comparisons with AQS security as an independent variable and attention and concentration battery scores as the dependent variables**

Measures	More secure children		Less secure children		***t***	***95% CI***	
	***M***	***SD***	***M***	***SD***		***LL***	***UL***
**Reaction time**							
Correct	27.93	4.59	29.38	1.09	-1.59	-3.24	.35
Time	.45	.24	.29	.26	2.94*	.05	.26
**Speed and accuracy**							
Correct	8.20	9.77	4.62	6.02	1.79	-.37	7.54
Time	.71	.75	.71	.79	-.02	-.33	.32
**Auditory recognition**							
Correct	7.54	1.70	6.77	1.70	2.08*	.03	1.50
Time	.87	.30	1.11	.29	-3.67**	-.36	-.11
**Visual recognition**							
Correct	7.94	1.60	6.85	2.55	2.76*	.31	1.87
Time	.73	.18	.81	.35	-1.79	-.18	.008
**Visual spatial recognition**							
Correct	8.83	2.62	7.42	2.87	2.42*	.25	2.55
Time	.61	.19	.70	.24	-1.95	-.17	.0010
**Digit span –forward**	1.32	3.42	.85	1.37	.69	-.88	1.82
**Digit span backward**	.36	.73	.20	.50	.85	-.17	.43
**Divided attention**							
Correct	5.90	2.42	3.85	2.85	3.77**	.97	3.13
Time	.95	.38	.81	.47	1.52	-.03	.30
**Multiple search letter**							
Correct	5.98	2.89	6.88	2.51	-1.46	-2.11	.31
Time	104.81	64.80	161.51	64.76	-4.02**	-84.57	-28.82
**Multiple search simbol**							
Correct	4.50	2.97	4.58	3.22	-.12	-1.38	1.21
Time	116.53	70.39	162.05	56.07	-3.07**	-74.77	-16.27

Note: correct: correct answers score; time: response times score; *CI* = confidence interval of the difference; *LL* = lower limit; *UL*= upper limit. *p < .05. **p < .001.

### Correlation analyses

A correlation matrix was performed. Table 3 shows the Pearson Correlation Coefficients, corrected for multiple comparisons. The Pearson Correlation matrix showed that “AQS criterion sort” scores were correlated with “auditory recognition” (time score), “visual recognition”, “divided attention”, and “voluntary shifting” scores. This finding suggests a relationship between attachment security and attention skills. As expected, significant correlations were found between the different “Attention and Concentration” battery scores. These measures assess domains that are strongly related to each other.

**Table 3:** **Pearson correlation matrix (all measures)**

	1	2	3	4	5	6	7	8	9	10	11	12	13	14	15	16	17	18	19
1. Reaction time - correct																			
2. Reaction time - time	.010																		
3. Speed and accuracy - correct	.04	.04																	
4. Speed and accuracy - time	-.01	.17^*^	.79^**^																
5. Auditory recognition - correct	-.04	.24^**^	.37^**^	.36^**^															
6. Auditory recognition - time	.01	.18^*^	-.33^**^	-.28^**^	-.14														
7. Visual recognition - correct	-.20^*^	.20^*^	.33^**^	.34^**^	.31^**^	-.22^**^													
8. Visual recognition - time	.00	.03	-.33^**^	-.28^**^	-.29^**^	.41^**^	-.41^**^												
9. Visual spatial recognition - correct	-.18^*^	.10	.47^**^	.41^**^	.35^**^	-.04	.38^**^	-.38^**^											
10. Visual-spatial recognition - time	.01	.11	-.42^**^	-.35^**^	-.16	.39^**^	-.15	.39^**^	-.52^**^										
11. Digit span forward	-.06	-.03	.44^**^	.36^**^	.25^**^	-.18^*^	.20^*^	-.20^*^	.30^**^	-.23^**^									
12. Digit span backward	-.14	.13	.57^**^	.46^**^	.24^**^	-.21^**^	.24^**^	-.21^*^	.32^**^	-.11	.31^**^								
13. Divided attention - correct.	-.09	.30^**^	.46^**^	.44^**^	.46^**^	-.20^*^	.48^**^	-.41^**^	.41^**^	-.15	.28^**^	.42^**^							
14. Divided attention - time	.20^*^	.06	-.05	-.08	.13	-.00	-.30^**^	-.07	-.05	.06	-.09	.00	.07						
15. Multiple search – letter - correct.	-.23^**^	.00	.34^**^	.35^**^	.16	-.06	.30^**^	-.06	.31^**^	-.17^*^	.25^**^	.20^*^	.23^**^	-.22^**^					
16. Multiple earch letter - time	-.13	-.28^**^	-.27^**^	-.19^*^	-.27^**^	.18^*^	-.15	.35^**^	-.22^**^	.30^**^	-.17^*^	-.07	-.32^**^	-.13	.35^**^				
17. Multple seach – simbol - correct	-.09	-.20^*^	.33^**^	.27^**^	.23^**^	-.25^**^	.43^**^	-.12	.36^**^	-.31^**^	.14	.08	.13	-.15	.49^**^	.02			
18. Multiple search simbol - time	.02	-.26^**^	-.19^*^	-.16^*^	-.16^*^	.16^*^	.04	.36^**^	-.13	.09	-.14	-.26^**^	-.24^**^	-.10	.24^**^	.49^**^	.54^**^		
19. AQS	.04	.09	.00	-.08	.12	-.31^**^	.09	-.20	.07	-.13	.00	-.04	.19	.11	-.11	-.17	-.04	-.10	

Note: correct: correct answers scores; time: response time scores. *p < .01. **p < .001.

### Regression analyses

Multiple regression analyses using “AQS criterion sort” scores, “gender”, and “age” as independent variables and “Attention and Concentration” battery scores as dependent variables were also conducted. Blockwise entry method was used. Multiple regression analyses look at theoretically meaningful associations between the various constructs controlling for relevant background variables (age, sex). Results showed the significant contribution to the model of all the variables (“AQS criterion sort” scores, “age,” and “sex”) AQS scores were significant predictors for “speed and accuracy” (response times scores; Multiple *R* = .79 , *F* = 174.59, df: 3, sig: *p* < .001), “auditory recognition” (correct answers scores: Multiple *R* = .48, *F =* 13.60, sig.: *p* < . 001, df. 3; response time scores: Multiple: *R* = .42, *F* = 10.12, sig: *p* < .001, df: 3); “visual recognition” (response time scores: Multiple *R* = .41, *F* = 9.36, *p* < .001, df. 3), “divided Attention” (correct answers scores: Multiple *R* = .58, *F* = 23.82, *p* < .001, df. 3; see Table 4).

**Table 4:** **Multiple regressions analyses using “AQS criterion sort” scores, “gender” and “age” as the independent variables and “Speed and accuracy,” “Auditory and visual recognition” and “Divided Attention” scores as the dependent variables**

Dependent variables	Speed and accuracy (response time scores)			Auditory recognition (correct answers scores)			Auditory recognition (response times scores)			Visual recognition (response times scores			Divided attention (correct answers scores)		
	Multiple ***R***: .79			Multiple ***R***: .48			Multiple: ***R*** .42			Multiple ***R*** : .41			Multiple ***R***: .58		
	***F*** = 174.59			***F =*** 13.60			***F*** = 10.12			***F*** = 9.36			***F*** = 23.82		
Independent variables	Unstandardized coefficient		***t***	Unstandardized coefficient		***t***	Unstandardized coefficient		***t***	Unstandardized coefficient		***t***	Unstandardized coefficient		*t*
	B	Std error		B	Std error		B	Std error		B	Std error		B	Std error	
AQS	-.51	.28	-2.37*	2.22	.96	.02*	-.66	.18	-3.68**	-.36	.13	-2.80*	.23		3.20*
Sex	.01	.06	.25	-.69	.27	.01*	.02	.05	-.44	-.04	.03	1.24	-.13		-1.83
Age	1.33	.05	22.76**	1.46	.25	5.64**	-.18	.04	-.37.**	-.15	.03	-4.47**	.54		7.77**

Note: * *p* < .05. ** *p* < .001.

Considering the developmental characteristics of the skills measured by the “Attention and Concentration” battery, a greater contribution of the variable “age” with respect to the variable “AQS criterion sort” scores is expected. Moreover, is well-known that sex influences attentional performances. However, the variable “AQS criterion sort” scores was a useful predictor for “speed and accuracy” (times), “auditory recognition” (correct responses and times), “visual recognition” (time), and “divided attention” (correct responses) scores.

## Discussion

The present study highlights several interesting points concerning the relationships that occur among attachment to preschool teachers and attention. Preliminary sex and age difference analyses showed males and females differ with regard to reaction times, in which females outperformed males, and with regard to visual spatial selectivity and maintenance, in which male outperformed female. These data were consistent with the scientific literature that has found that males and females perform differently in some cognitive domains (Kaufman 2007; Terlecki and Newcombe 2005; Voyer et al. 1995). As expected, significant age related differences were also observed in the current study. With increasing age, attention skills improve, allowing on-task focus and improved performance (Plude et al. 1994).

With regard to the principal aim of this research, the findings showed that child/preschool teacher attachment is related to attentional performance. This observation confirms the hypothesis that socio-emotional development could significantly contribute to influence attentional skills (Gillath, Giesbrecht, and Shaver, Gillath et al. 2009; Silva et al. 2012). Children who are more securely attached to their professional caregivers and obtaining an “AQS criterion sort” score that indicates they are well-adjusted to preschool had better performance on most of the “Attention and Concentration” tasks. In particular, children with more secure attachments presented better reaction time and better auditory, visual, and visual spatial selectivity and maintenance as compared to their peers with less secure attachment. Moreover, they obtained higher performances in the tests assessing “divided attention” and “voluntary shifting,” compared with children with less secure attachment. Likewise, previous studies have shown that the quality of emotional interactions may predict academic achievement trajectories, and increasing teacher-child interactions may facilitate child cognitive performances (Bergin and Bergin 2009; Pianta et al. 2008).

A positive bond between children and their teachers probably encourages children to direct, maintain and distribute their attention correctly, increasing their general cognitive performances. In this regards, it is important to consider that the relationship between attachment and cognitive abilities is bidirectional. Attention skills support the child relationship with adult and peers, and encourage his social-emotional adjustment, ameliorating child’s ability to understand and copy the behavioral demands of the school. Children with high attention skills may be able to distribute visual attention spatially, deploy attention over time, and allocate attention to visual objects. This increases their ability to recognize the significant aspects of a situation, and the ability to respond to the requests of the teachers. Thus, teachers find it easier to respond, react sensitively and create secure relationships with these children. For this reason, attachment has at least two functions pertinent to classrooms: providing feelings of security and socializing with other children (Bergin and Bergin 2009).

The correlation analyses support these considerations. Correlation analysis showed that the security of attachment is correlated with visual and auditory selectivity and maintenance, divided attention, and voluntary shifting. The relationship among attachment security and attentional skills confirms the numerous observations that positive child–teacher relationships could increase school adjustment and success (Hamre and Pianta 2001; Mashburn et al. 2008; Pianta, Steinberg and Rollins, 1995). Attention underlies all basic and complex cognitive activity, such as those involved in learning, memory and problem solving. The significant correlations between quality of attachment and attention skills highlighted the “power” of a positive child/teacher relationship into cognitive performance.

Finally, multiple regression analyses showed that attachment to professional caregivers was significantly related to the attention skills. Particularly, the results showed that “AQS criterion sort” scores were significant predictors of the reaction times related to a choice, of the visual and auditory selectivity and maintenance, and of the divided attention. As expected, “age” and “sex” were significant predictors for many Attention and Concentration battery scores. It is predictable that children of different ages present different attentional performances, and is known that male and female present differences in attention skills. However, even if age and sex are well-known variables in determining cognitive development, the quality of attachment influences attention significantly. Interestingly, attachment security is a better predictor with respect to “sex” for reaction times related to a choice, auditory and visual maintenance, and divided attention.

Although the nature of my data does not allow me to formulate a directional hypothesis, this last issue is of high interest because current theories emphasize the role that socio-emotional variables play in cognitive performances and in school achievement. An early assessment of the quality of the relationship between children and their preschool teachers might assist in identifying children who are not well-adapted to preschool, with the aim to improve the quality of their socio-emotional adjustment. This could have positive consequences in the children cognitive performances.

## Conclusions

The results of the present study revealed that children’s secure base behaviour, in relationship with their preschool teachers, is related to attention skills. Considering the role of attention skills in all cognitive and behavioural performances, a non-secure child/teacher bond might reduce children’s participation in collaborative learning activities and influence scholastic achievement adversely (Sthulman and Pianta 2004). Compared with their more securely attached peers, children with less secure attachments to their teachers presented slower reaction time, and lower auditory, visual and visual spatial selectivity and maintenance. For these reasons, these children might be less able to spontaneously exploit the learning occasions offered to them in the social and physical environments of preschool.

The findings of this study might also have clinical relevance. Attachment to a teacher can be easily measured in preschool, and its assessment might allow us to identify children who are able to benefit from the social and cognitive stimuli that increase their attention skills, explorative behaviour, and environmental adaptability. Indeed, all of these aspects are connected to the development of cognitive and behavioural skills. In particular, attention plays a pivotal role in the effectiveness of each mental process, thus energizing the activity of the cognitive functions. In conclusion, the precocious assessment of the child/preschool teacher relationships might reveal a relationship pattern that hinders the optimization of their learning occasions.

### Study limitations and future directions

This study has several limitations. First, caution needs to be taken when interpreting my findings. Indeed, the relationships between attachment and attention are complex and difficult to examine. In addition, the use of an observational instrument has some well known limitations, and observer effects may occur. However age of participants and the characteristics of the preschool setting make these measures appropriate for this study.

Despite these limitations, the present study suggests interesting applications. These findings emphasize the potential usefulness of screening all preschoolers and kindergarteners for potential behavioural and emotional problems. The early identification of insecurely attached children and the adoption of subsequent prompt and effective measures may increase their attentional functioning and may be critical to prevent scholastic difficulties. Intensive prevention trials centred on social and emotional skills are relatively inexpensive and could easily be realized in school.
